# PCNA in Cervical Intraepithelial Neoplasia and Cervical Cancer: An Interaction Network Analysis of Differentially Expressed Genes

**DOI:** 10.3389/fonc.2021.779042

**Published:** 2021-11-26

**Authors:** Panagiotis Giannos, Konstantinos S. Kechagias, Sarah Bowden, Neha Tabassum, Maria Paraskevaidi, Maria Kyrgiou

**Affiliations:** ^1^ Society of Meta-Research and Biomedical Innovation, Cancer Research Working Group, London, United Kingdom; ^2^ Department of Life Sciences, Faculty of Natural Sciences, Imperial College London, London, United Kingdom; ^3^ Department of Metabolism, Digestion and Reproduction, Faculty of Medicine, Imperial College London, London, United Kingdom; ^4^ Department of Obstetrics and Gynaecology, Chelsea and Westminster Hospital National Health Service (NHS) Foundation Trust, London, United Kingdom; ^5^ Institute of Reproductive and Developmental Biology, Imperial College London, London, United Kingdom; ^6^ Department of Surgery and Cancer, Faculty of Medicine, Imperial College London, London, United Kingdom; ^7^ West London Gynaecological Cancer Centre, Imperial College Healthcare National Health Service (NHS) Trust, London, United Kingdom

**Keywords:** cervical intraepithelial neoplasia, CIN, cervical cancer, gene biomarkers, cervical disease

## Abstract

The investigation of differentially expressed genes (DEGs) and their interactome could provide valuable insights for the development of markers to optimize cervical intraepithelial neoplasia (CIN) screening and treatment. This study investigated patients with cervical disease to identify gene markers whose dysregulated expression and protein interaction interface were linked with CIN and cervical cancer (CC). Literature search of microarray datasets containing cervical epithelial samples was conducted in Gene Expression Omnibus and Pubmed/Medline from inception until March 2021. Retrieved DEGs were used to construct two protein-protein interaction (PPI) networks. Module DEGs that overlapped between CIN and CC samples, were ranked based on 11 topological algorithms. The highest-ranked hub gene was retrieved and its correlation with prognosis, tissue expression and tumor purity in patients with CC, was evaluated. Screening of the literature yielded 9 microarray datasets (GSE7803, GSE27678, GSE63514, GSE6791, GSE9750, GSE29570, GSE39001, GSE63678, GSE67522). Two PPI networks from CIN and CC samples were constructed and consisted of 1704 and 3748 DEGs along 21393 and 79828 interactions, respectively. Two gene clusters were retrieved in the CIN network and three in the CC network. Multi-algorithmic topological analysis revealed PCNA as the highest ranked hub gene between the two networks, both in terms of expression and interactions. Further analysis revealed that while PCNA was overexpressed in CC tissues, it was correlated with favorable prognosis (log-rank P=0.022, HR=0.58) and tumor purity (P=9.86 × 10^-4^, partial rho=0.197) in CC patients. This study identified that cervical PCNA exhibited multi-algorithmic topological significance among DEGs from CIN and CC samples. Overall, PCNA may serve as a potential gene marker of CIN progression. Experimental validation is necessary to examine its value in patients with cervical disease.

## Introduction

Cervical cancer (CC) constitutes one of the most commonly diagnosed gynecological cancers worldwide (1). Progression of CC is characterized by the transition from an initial premalignant state called cervical intraepithelial neoplasia (CIN), that is graded based on the extension of dysplastic abnormalities in the epithelial cells of the cervix. Three stages of pre-malignancy can be defined: CIN-I [low-grade intraepithelial lesion (LSIL)], CIN-II and CIN-III [high-grade intraepithelial lesion (HSIL)] (2, 3). Persistent infection with high-risk human papilloma virus (HPV) is considered necessary for the development of CIN, however the majority of women clear the infection (2, 3). Nevertheless, a fraction of women develop CIN that can progress to CC if not detected and treated (2, 3).

The prolonged period necessary for progression from carcinogenic HPV infection to precancerous CIN to cancer, allows for detection and treatment of these lesions and dramatic reductions in mortality from cancer (4). However, concerns related to the low accuracy of Papanicolaou test and the financial burden pertained to cytological-based screening, have been raised (5). Additionally, overtreatment for CIN remains a matter of considerable discussion (6). Thus, markers for estimating the progression of CIN may be of potential value in the optimization of cervical screening and treatment.

Genetic markers of CIN and CC are poorly defined, but genetic variation likely plays a major role in the different host-viral interactions observed across individuals infected with HPV (7). Cervical carcinogenesis, likely depends on a complex interaction between HPV exposure, genetic predisposition due to inheritance of common risk variants, exposure to further environmental carcinogens and progressive carcinogenic processes such as loss of tumor suppressor genes and apoptotic dysregulation (7, 8). This phenomenon also requires the dynamic interaction of multiple strongly associated genes, as opposed to the dysregulated expression of individual ones. Thus, analysis of differentially expressed genes (DEGs) and their interactome, could be pivotal in the development of markers for the monitoring of CIN progression. Our study focused on examining cervical epithelial gene expression from patients with CIN and CC. The aim was to identify potential gene biomarkers whose dysregulated expression and protein interaction interface were involved in CIN and CC.

## Materials and Methods

Our study focused on the identification of genes with a potential role in the progression of pre-cancerous cervical lesions to cervical cancer.

### Collection of Microarray Data

The screening of the literature was conducted from inception until March 2021. We initially searched the National Center for Biotechnology Information (NCBI) Gene Expression Omnibus (GEO) and the search terms included: (cervix OR cervical). Additionally, we performed a search of the National Library of Medicine (NLM) PubMed using the terms: [(differentially expressed genes) AND (cervix OR cervical)]. Two authors (PG and KSK) created the search strategy and conducted the screening of the yielded datasets. Discrepancies in the literature search process were discussed and resolved by MK.

Datasets were restricted based on organism type (*Homo sapiens*), expression profiling (microarray), sample type (cervical epithelial tissue) and disease state (CIN and CC). No restrictions in terms of language and geographic region were used for dataset retrieval. Datasets lacking expression data for controls were excluded. No exclusion criteria related to the baseline characteristics of patients from which tissue sections were obtained, were applied.

### Identification of Differentially Expressed Genes

Cervical epithelial samples from healthy controls were compared to those with CIN or CC and DEGs were identified using ImaGEO (9). Integration of differential gene expression was performed using the random effect model and genes with the strongest average effect across the collected datasets were retrieved. DEGs with P<0.05 corrected by the Benjamini-Hochberg False Discovery Rate were considered as significant. DEGs with *Z* score>1.96 were regarded as upregulated, while those with *Z* score<1.96 as downregulated (corresponding to a 5% significance level).

### Construction of Protein-Protein Interaction Networks

DEGs from CIN and CC were used separately to construct two networks of encoded proteins using The Search Tool for the Retrieval of Interacting Genes (STRING) (10). The predicted protein-protein interaction (PPI) networks were identified using a medium probabilistic confidence score of >0.4 and mapped with Cytoscape (11). The purpose of applying a reasonably moderate cut-off score was to increase the coverage of all possible protein interactions without overestimating their precision. Non-interacting proteins were excluded from the networks.

### Identification of Clustering Modules and Hub Genes

Highly interconnected clusters or modules in the PPI networks were identified using the Molecular Complex Detection (MCODE) (12). Selection of cut-off was ensued following manual inspection of clusters and a score resulting in the distinct segregation of clusters into groups was considered. Clusters with MCODE score >20 were regarded as significant modules.

The interactions of module DEGs in the PPI networks were analysed using CytoHubba (13). Module DEGs were ranked based on the intersection of 11 established topological algorithms as described by Chin et al., namely: Degree, Closeness, Betweenness, Radiality, Stress, EcCentricity, BottleNeck, Edge Percolated Component (EPC), Maximum Neighborhood Component (MNC), Density of Maximum Neighborhood Component (DMNC) and Maximal Clique Centrality (MCC) (13). The top 10 ranked module DEGs that overlapped in the CIN and CC networks, were considered as hub genes.

### Analysis of Prognosis, Expression Level, and Tumor Purity of Hub Genes in CC

The highest ranked hub gene, both in terms of expression and interactions, was retrieved as a potential gene biomarker in CIN progression and was further characterized. Firstly, using publicly available transcriptome data from The Cancer Genome Atlas (TCGA) via the Gene Expression Profiling Interactive Analysis 2 (GEPIA2), its differential expression in CC tissues based on a P<0.05 and |log_2_ fold change|>2, was assessed (14). Secondly, its prognostic value in patients with CC and controls, which were divided into high and low expression groups, was examined. Correlation with overall survival (OS) and disease-free survival (DFS) were established using a log-rank P<0.05. Thirdly, its association between expression and the tumor microenvironment in terms of purity, was estimated in CC using the partial Spearman’s correlation (partial rho) *via* the Tumor Immune Estimation Resource 2 (TIMER2) algorithm (15).

## Results

### Overview of Microarray Datasets

**Figure 1:**
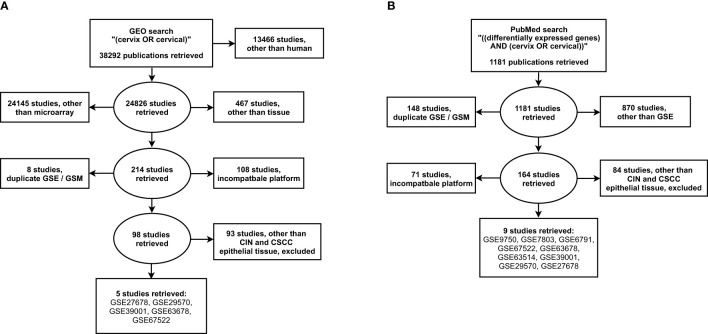
Search strategy for the selection of eligible gene expression studies from the **(A)** NCBI GEO and the **(B)** NLM PubMed. GSE, gene expression series; GSM, gene expression sample. NCBI, National Center for Biotechnology Information; GEO, Gene Expression Omnibus; NLM, National Library of Medicine.

Our search of the GEO database resulted in 38292 datasets, of which 24826 were categorized as human. From these, 214 microarray tissue-containing datasets were retrieved and 116 datasets with either duplicate gene expression sample and series or incompatible platforms, were excluded. Screening of the resulting 98 datasets, based on cancer type and tissue type, revealed one CIN [GSE27678 (16–18)] and five CC datasets [GSE27678, GSE29570 (19), GSE39001 (20), GSE63678 (21), GSE67522 (22, 23)] with cervical epithelial samples (**Figure 1A**).

Our additional search of the PubMed database, resulted in 1181 datasets, of which 311 corresponded to gene expression studies. From these, 219 microarray datasets with either duplicate gene expression sample and series or incompatible platform were excluded. Screening of the resulting 164 datasets, resulted in three CIN [GSE7803 (24), GSE27678, GSE63514 (25)] and nine CC datasets [GSE6791 (26), GSE7803, GSE9750 (27), GSE27678, GSE29570, GSE39001, GSE63514, GSE63678, GSE67522] with cervical epithelial samples (**Figure 1B**).

The overlap between the two literature searches ultimately revealed three CIN (GSE7803, GSE27678, GSE63514) and nine CC datasets (GSE6791, GSE7803, GSE9750, GSE27678, GSE29570, GSE39001, GSE63514, GSE63678, GSE67522). The retrieved datasets included cervical epithelial tissue biopsies from healthy controls (n=130) and patients with either CIN (n=115) or CC (n=218). From the identified CC datasets, four included squamous cell carcicnomas (GSE7803, GSE27678, GSE63514, GSE67522) and three included both squamous cell carcinomas and adenocarcinomas (GSE9750, GSE29570, GSE39001), while in two the histopathology of CC was not specified (GSE6791, GSE63678).

### Differentially Expressed Genes in CIN and CC

**Figure 2:**
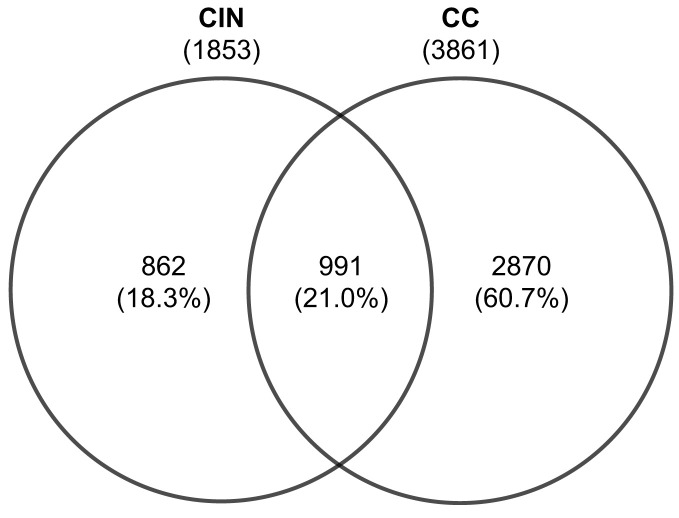
Venn diagram of the differentially expressed genes from cervical epithelial samples in patients with CIN and CC. Values are numbers unless otherwise stated. CC, Cervical cancer; CIN, Cervical intraepithelial neoplasia.

Integration of differential gene expression across the retrieved datasets revealed 1853 DEGs in CIN patients, when compared to healthy controls. Of these, 1157 DEGs were upregulated, and 696 were downregulated. Further, a total of 3861 DEGs were identified in CC patients when compared to healthy controls. Of which, 1986 were upregulated and 1875 were downregulated. Comparative analysis between these expression profiles revealed 991 overlapping DEGs, 862 unique to CIN and 2870 to CC (**Figure 2**).

### Protein-Protein Interaction Networks and Modules in CIN and CC

**Figure 3:**
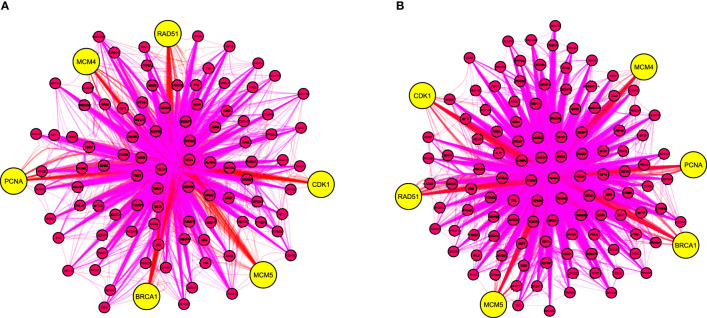
The top 10 overlapping hub genes of clustering modules in the protein-protein interaction network of differentially expressed genes from **(A)** CIN and **(B)** CC patients. Yellow nodes indicate hub genes. BRCA1, BRCA1 DNA repair associated; CIN, cervical intraepithelial neoplasia; CC, cervical cancer; CDK1, cyclin dependent kinase 1; MCM4, minichromosome maintenance complex component 4; MCM5, minichromosome maintenance complex component 5; PCNA, proliferating cell nuclear antigen; RAD51, RAD51 recombinase.

Two PPI networks of DEGs from CIN and CC datasets were constructed and consisted a total of 1704 and 3748 DEGs along 21393 and 79828 interactions, respectively (**Figures S1A**, **S2A**). Two highly interconnected modules were identified in the CIN network and three in the CC network (**Figures S1B**, **S2B** and **Tables S1**, **S2**). From the top 10 ranked hub module DEGs in each network, six overlapping genes were revealed: PCNA (proliferating cell nuclear antigen), CDK1 (cyclin dependent kinase 1), MCM4 (minichromosome maintenance complex component 4), BRCA1 (BRCA1 DNA repair associated), MCM5 (minichromosome maintenance complex component 5) and RAD51 (RAD51 recombinase) (**Figure 3**, **Table 1**, **Tables S3**, **S4**).

**Table 1:** The top ranked and overlapping hub genes according to 11 topological algorithms in the protein-protein interaction (PPI) networks of cervical intraepithelial neoplasia (CIN) and cervical cancer (CC) differentially expressed genes.

		CIN		CC	
ID	Name	FDR	Z-Score*	FDR	Z-Score*
CDK1	Cyclin dependent kinase 1	5.38 x 10^-7^	5.86	0.00	8.80
MCM5	Minichromosome maintenance complex component 5	1.65 x 10^-4^	4.56	6.69 x 10^-10^	6.67
BRCA1	BRCA1 DNA repair associated	7.54 x 10^-8^	6.26	0.00	9.51
PCNA	Proliferating cell nuclear antigen	3.86 x 10^-12^	7.94	2.32 x 10^-11^	7.19
MCM4	Minichromosome maintenance complex component 4	7.97 x 10^-10^	7.06	1.48 x 10^-9^	6.54
RAD51	RAD51 recombinase	8.77 x 10^-11^	7.45	4.46 x 10^-7^	5.54

FDR, False discovery rate.

*Expression level compared to healthy controls, following P < 0.05 corrected by Benjamini-Hochberg False Discovery Rate.

### Prognosis, Expression Level, and Tumor Purity of PCNA in CC

**Figure 4:**
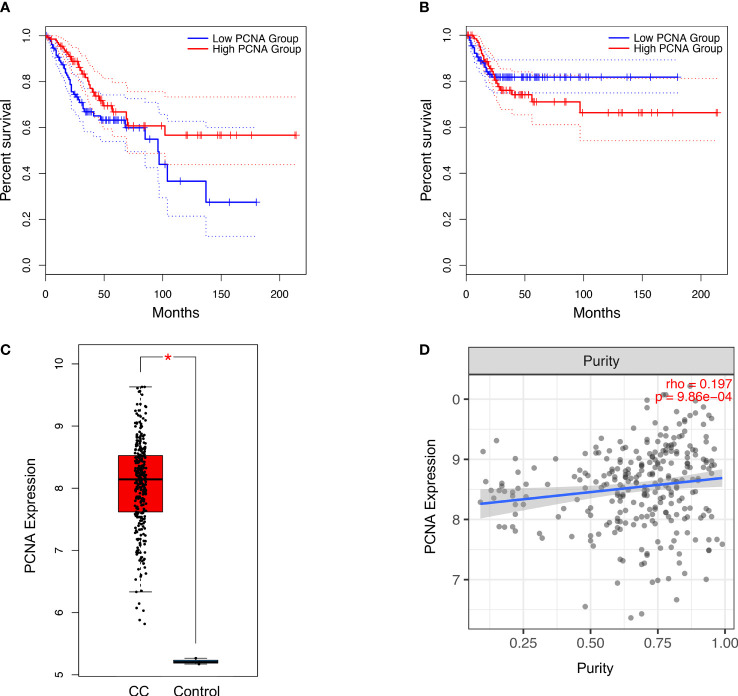
Association of PCNA expression with **(A)** overall survival (log-rank P=0.022, n(high)=146, n(low)=146) and **(B)** disease-free survival (log-rank P =0.55, n(high)=146, n(low)=146) in CC patients. Significance was determined using a log-rank P<0.05. Overall expression **(C)** (transcripts per million) of PCNA based on the TCGA in patients with CC. Significance was determined using a P<0.05 and |log_2_ fold change|>2. Association between expression level of PCNA and **(D)** tumour purity in patients with CC. Estimation was determined using the partial Spearman’s correlation (rho). CC, Cervical Cancer; PCNA, Proliferating cell nuclear antigen; TCGA, The Cancer Genome Atlas. *P < 0.05.

Survival analysis of the TCGA (309 patients) revealed that low expression of PCNA correlated with significantly reduced OS (log-rank P=0.022, HR=0.58) in CC patients, but not with DFS (log-rank P=0.55, HR=1.2) (**Figures 4A, B**). Expression of PCNA was significantly upregulated in CC tissues when compared to normal (**Figure 4C**). Pan-cancer analysis demonstrated that dysregulated expression of PCNA was highest in CC among 24 other tumor types (**Figure S3**). Additionally, the expression level of PCNA was significantly and positively correlated with tumor purity in CC (P=9.86 × 10^-4^, partial rho=0.197) (**Figure 4D**).

## Discussion

Analysis of differentially expressed genes from cervical epithelial samples of CIN and CC patients, identified two gene modules in the CIN network and three in the CC network. Multi-algorithmic topological analysis revealed six overlapping hub genes, namely: PCNA, CDK1, MCM4, BRCA1, MCM5 and RAD51. PCNA was the highest ranked hub gene both in terms of expression and interactions, rendering its potential value as a marker of CIN progression.

### Findings in the Context of the Literature

PCNA encodes a nuclear protein that is maximally expressed in late G1 and S phases of the cell cycle (28–32). It constitutes a core component of the replication and repair machinery, acting as an auxiliary yet orchestral co-factor of the DNA polymerase δ and ϵ (32–35). By encircling the DNA in a “sliding clamp” formation and recruiting crucial factors to the replication fork, PCNA increases the processivity of the polymerase during replication (32–35). Its role in tumor initiation and progression is linked with perturbed dynamics of DNA synthesis and post-replicative repair, which are all driven from its dysregulated activation (36–38).

A plethora of experimental studies have investigated PCNA expression in CIN and CC (32, 39). Earlier reports have demonstrated significant and positive correlation of PCNA expression with mitotic index and tumor grade (40–44). Additionally, PCNA expression was found to be associated with the presence of oncogenic HPV, possibly due to the suboptimal interaction of the HPV oncoprotein E7 with p21^Cip1/Waf1^ which physiologically results in PCNA underexpression (45, 46). However, PCNA expression has shown major upregulation upon CIN3 progression and further invasiveness, irrespective of HPV status (47). Therefore, it may be speculated that overexpression of PCNA is primarily associated with CIN progression and to a lesser extent with HPV infection which has a more prominent role in CIN onset.

In spite of being a strong marker of cell proliferation and CIN progression, the prognostic value of PCNA in CC has not been systematically studied and remains a matter of considerable debate (45, 48–50). While PCNA expression did not exhibit prognostic value in two reports by Branca et al. and Costa et al., another study which included 111 CC patients demonstrated association between its overexpression and decreased survival (32, 45, 51). Conversely and in accordance with our findings, a more recent *in silico* study showed that high expression of PCNA was rather associated with favorable prognosis, although this result was based on the analysis of only 300 CC patients (52). Taken together, the role of PCNA in the prediction of CC prognosis remains inconclusive.

### Strengths and Limitations

This is the first study that comprehensively examined the protentional role of DEGs and their interactome as gene biomarkers in CIN progression, using 9 publicly available datasets with more than 450 included patients. In doing so, we employed a multi-algorithmic protein-interaction based approach that relied on different levels of filtering.

Our study also had limitations. Cervical epithelial samples from CIN patients were not limited to any specific HPV infection status. Inclusion of a heterogeneous genotypic distribution of HPV prevalence and type among CIN patients, restricts the predictive value of PCNA as a potential marker of CIN progression. However, HPV types share a common genomic organization and thus the number and the different combinations of encoded oncoproteins contributing to the malignant initiation and transformation, are believed to be similar (53). Additionally, it was not possible to control for other potential confounders such as demographic characteristics (e.g. age) and medical comorbidities (e.g. obesity) in included patients, which could have resulted in residual confounding (54).

Included studies employed different expression profiling platforms, a confounding factor known to hinder statistical power and reliability in detection of DEGs - this type of heterogeneity often results in different expression scales that inevitably reduce the number of integrated DEGs, even after normalization (55). We partially addressed unknown cross-study heterogeneity by employing a random effect model for establishing the significance of DEGs between studies (55–59).

## Conclusions

The disease burden of CC has significantly decreased in recent years in developed countries, however the financial costs of screening, the limited capacity of cytological-based diagnosis and the competing risk of reproductive consequences following treatment, remain a challenge. Our study identified that cervical PCNA exhibited multi-algorithmic topological significance among DEGs from CIN and CC samples. Overall, PCNA may serve as a potential gene marker of CIN progression. Experimental validation is necessary to examine the screening, diagnostic and prognostic value of PCNA in patients with CIN and CC.

## Data Availability Statement

Publicly available datasets were analyzed in this study. This data can be found at the Biotechnology Information (NCBI) Gene Expression Omnibus (GEO) using the following accession numbers: GSE7803, GSE27678, GSE63514, GSE6791, GSE9750, GSE29570, GSE39001, GSE63678, GSE67522.

## Author Contributions

The study was conceived and designed by PG and KSK. The data was acquired and collated by PG and KSK, and analyzed by PG. The manuscript was drafted and revised critically for important intellectual content by all authors. All authors gave final approval of the version to be published and have contributed to the manuscript.

## Conflict of Interest

The authors declare that the research was conducted in the absence of any commercial or financial relationships that could be construed as a potential conflict of interest.

## Publisher’s Note

All claims expressed in this article are solely those of the authors and do not necessarily represent those of their affiliated organizations, or those of the publisher, the editors and the reviewers. Any product that may be evaluated in this article, or claim that may be made by its manufacturer, is not guaranteed or endorsed by the publisher.
