# Supplementary material for: PCNA in Cervical Intraepithelial Neoplasia and Cervical Cancer: An Interaction Network Analysis of Differentially Expressed Genes
Source: Front Oncol. 2021 Nov 26;11:779042. doi: 10.3389/fonc.2021.779042 (PMC8661029; doi:10.3389/fonc.2021.779042)
Supplement: Supplementary file 1 [file DataSheet_1.docx]

**Supplementary Tables**

| **Table S1.** The top clustering modules according to Molecular Complex Detection (MCODE) in the protein-protein interaction (PPI) networks of cervical intraepithelial neoplasia (CIN) differentially expressed genes. | | | |
| --- | --- | --- | --- |
|  | | | |
| **Module** | **MCODE Score*** | **Gene Count** | **Genes** |
| Module 1 | 85.163 | 99 | KIF4A, NCAPG, MCM6, DTL, HJURP, TIMELESS, RRM2, HELLS, PTTG1, CDK1, STIL, FOXM1, CCNE2, CENPA, RAD51AP1, KIF2C, CDCA8, SPDL1, CCNB2, TTK, CDC20, KIF20B, MYBL2, CKS2, SMC2, NUSAP1, FANCI, FBXO5, KIF11, KIF14, TOP2A, POLE2, MKI67, RACGAP1, MELK, CDC7, HMMR, TK1, MAD2L1, BIRC5, CENPN, DEPDC1, CENPF, PCNA, KIF20A, RRM1, KIF22, ATAD2, TPX2, ERCC6L, CDKN3, SHCBP1, BRCA1, DLGAP5, CDC25C, CCNB1, RFC4, FEN1, PRIM1, KNTC1, NCAPG2, CKS1B, KPNA2, TRA, SMC4, EZH2, CDC6, TACC3, ZWINT, OIP5, RAD51, RAD54L, CEP55, CHAF1A, PRC1, ECT2, NDC80, CENPM, CKAP2, MCM5, KIF23, SPAG5, MCM3, ASPM, MCM4, MCM7, KIF15, CDC25A, SKA1, PCLAF, WDHD1, KIF18A, SPC25, AURKA, GINS2, NEK2, CHEK1, CDT1, GMNN |
| Module 2 | 23.789 | 58 | DNAJC9, SF3B3, MAGOHB, DHX16, TOP3A, PTBP1, DDX46, SNRPF, HNRNPD, GINS4, PSMA2, PSME1, AQR, CSTF1, SEM1, PSMB3, MIR7703, CSTF2, LSM6, CRNKL1, PAPOLA, DNAJC8, SNRPG, PARP1, PRPF6, UPF3B, CTNNBL1, PSMA4, HNRNPR, SNRPB2, LSM3, SRSF5, RECQL4, NCBP2, SYF2, PSMD7, GPKOW, SMC6, SRSF11, CSTF3, POLD3, PSMA3, PPIH, PSMB1, MCMBP, U2SURP, PSMC2, PAICS, POLR2K, MRE11, TFIP11, PSMD9, LSM5, POLR2D, PSMB9, LSM8, HAT1, PSMA5 |
| *Significance based on MCODE cluster score >20. | | | |

| **Table S2.** The top clustering modules according to Molecular Complex Detection (MCODE) in the protein-protein interaction (PPI) networks of cervical cancer (CC) differentially expressed genes. | | | | |
| --- | --- | --- | --- | --- |
|  | | | | |
| **Module** | **MCODE Score** | | **Count** | **Genes** |
| Module 1 | | 102.763 | 119 | ARHGAP11A, RRM2, POLE2, HMMR, TOP2A, DSCC1, CDKN3, HELLS, GINS2, UBE2C, NCAPD2, CCNB2, CHEK1, POLQ, DLGAP5, KIF18A, MCM6, CDT1, PCNA, MYBL2, KNTC1, KIF2C, SMC4, TYMS, PRIM1, NDC80, NCAPH, PLK1, GMNN, MCM5, RACGAP1, ESPL1, CDC6, TK1, KIF20B, BIRC5, PKMYT1, SMC2, BUB1B, AURKA, ASPM, NEK2, FBXO5, CDC20, FOXM1, ORC1, RAD54L, FANCI, SPAG5, BUB1, MAD2L1, CLSPN, CEP55, CKAP2, PBK, KIFC1, MCM7, CDK2, MCM10, CCNE2, CKS1B, KIF23, MCM3, SHCBP1, CENPN, CDC25A, BRCA1, GINS1, NCAPG2, KPNA2, KIF20A, KIF15, RFC4, KIF11, RAD51, CENPF, CCN1, PRC1, ZWINT, PIMREG, KIF22, ECT2, RFC3, CENPU, KIF14, MKI67, MELK, HJURP, FEN1, ZWILCH, MCM4, TIMELESS, RAD51AP1, ASF1B, CDCA8, CDC45, DEPDC1, PLK4, TROAP, OIP5, TPX2, EZH2, ERCC6L, AURKB, EXO1, RRM1, PCLAF, CENPM, ATAD2, PTTG1, TRIP13, TTK, WDHD1, NCAPG, CDC25C, CKS2, CDK1, NUSAP1, SPC25 |
| Module 2 | | 49.555 | 212 | NDEL1, CHAF1A, STAG1, HUWE1, NUDC, U2SURP, CENPI, HNRNPA1, CPSF4, HNRNPR, DHX16, LPAR2, SMC3, ADRA2A, NPY5R, PRPF3, SNRPD3, CCL16, PRKN, NUP160, BTBD1, ADCY2, POLR2K, CXCL1, PPIH, LSM2, CLP1, LSM5, RANGAP1, FBXO17, SF3A2, GLMN, SRSF6, SPC24, ELAVL1, LONRF1, CUL5, RNASEH2A, CXCR2, CCNE1, CSTF2, NUDT21, TFIP11, CHEK2, DDX23, HNRNPF, POLD1, RFC5, CXCL8, CXCL9, MIS12, ELOB, UBE2A, AGT, SF3B4, CXCL12, SRSF1, SRSF4, PUF60, LIG1, CDC5L, POLR2D, LSM6, TAS2R4, NEIL3, SKP2, GAN, TRIM37, NSL1, SNRPE, GPSM2, GPR18, CCNF, CENPQ, SNRPB, SF3B5, ITGB3BP, PJA1, SMC1A, UBE2E1, PRCC, GNB1, KLHL3, USP39, KLHL41, BUB3, CCR2, GNG5, POLA1, CCL21, MAGOHB, HTR1E, SPSB1, C3AR1, FBXL5, RNF19B, SNRPA1, AGTR2, RBM22, UBE2Q1, TRA2B, GNG7, PRPF31, HERC5, PSAP, GNB5, RNF220, DYNC1I1, NUP43, GNB3, E2F8, NCBP2, UBA52, MIS18BP1, UBE2L6, ZBTB16, RNF7, UBE3C, SNRPD2, KLHL2, SNRPC, SNRPA, PPP2R5A, LSM4, UBE2Z, CCR1, BTRC, POLE, POLR2H, CNR1, CXCL13, ADCY1, UBE2S, UBA5, KEAP1, LMNB1, DYNC1H1, UPF3B, ELAVL2, RBCK1, ASB6, RNF138, POLA2, RAD21, GALR3, CTNNBL1, CCL20, NDE1, CHAF1B, PPP2R1B, GABBR1, ASB13, HNRNPA2B1, CENPO, UBE2D4, KIF2A, STIL, CSTF1, LPAR3, SNRPG, CXCL11, RBM8A, RNF114, XPO1, SF3A3, CX3CR1, FBXW2, TRAIP, CDC23, LPAR1, RNF34, PRPF40A, NCBP1, APP, ASB7, SNW1, PRPF19, RNPS1, SNRPF, NUP133, SMURF2, SNRNP27, GNGT1, NMUR1, C5AR1, GNG11, DYNLL1, NUP85, ANXA1, DSN1, WBP11, CXCL2, SNRNP40, CXCL6, NEDD4L, PARPBP, C3, PRPF4, TAS2R8, TRIM36, FBXL7, BDKRB1, CKAP5, SYMPK, CSTF3, TAS2R13, PTGER3, P2RY4, S1PR1, CXCL10, UBA1 |
| Module 3 | | 31.315 | 150 | PLCB4, RSRC1, SNORA21, EDN2, SMC5, TIPIN, RPL27A, DNA2, PSMB2, MSLN, RPL3, PSMD14, DCP1A, TAC1, EEF2, RPS5, PSMD12, PSMA4, DONSON, ADAM10, ORC2, BARD1, RAD17, PSMB1, PSMB5, MRPS2, TACR1, STN1, SMG5, CALU, MRTO4, RPL31, CHRDL1, RPL14, CST3, OXTR, HTR2B, PIK3CA, PTGFR, PSMC1, PABPC1, DBF4, EDNRB, RPL23A, MXRA8, RPL34, GNRHR, PRKCSH, RPL19, BMP4, DUT, WFS1, ORC6, NMB, IGFBP4, UNG, PSMC4, QSOX1, F5, ORC5, RPA1, RPL10A, GFM1, NTS, GNA14, RPA2, GOLM1, PSMD2, TMEM132A, BMP15, SPARCL1, MRPL3, MRPS15, MRPL13, E2F1, MRPS11, LAMC1, RPL18, APOB, PSMA5, PSMA3, PSMB4, PSMB7, NMBR, PRSS23, TOPBP1, STC2, IGFBP7, AMELX, RPA3, PSMD4, TRH, RPL38, GAS6, HRC, ATR, LPAR6, PRIM2, MBTPS1, PIK3R3, PDIA6, AGTR1, RPL29, RPL22, IGFBP5, MSH2, EIF5A2, PSMB8, RPL15, HSP90B1, EDNRA, ATAD5, BRS3, SCG3, APOL1, PIK3R2, EDN3, MRPL11, SEC61A1, HCRTR2, HCRT, SLBP, RPL39L, GPC3, CDK4, RPL36, GRP, EIF2S3, ADRA1D, FSTL1, SEM1, PSMB3, MRPS12, RPS9, SRP54, RCN1, PIK3R1, RPL37A, DDX39A, RPS17, KALRN, RPL32, APOE, MRPL16, TRIO, LPAR4, RPS29, RPS25, SPP1, PSMB10, |
| *Significance based on MCODE cluster score >20. | | | | |

| **Table S3.** Top 10 hub genes according to 11 topological algorithms ranked in the protein-protein interaction (PPI) network of cervical intraepithelial neoplasia (CIN) differentially expressed genes. Numbers represent score. | | | | | | | | | | | | | | | | | | | | | | | |
| --- | --- | --- | --- | --- | --- | --- | --- | --- | --- | --- | --- | --- | --- | --- | --- | --- | --- | --- | --- | --- | --- | --- | --- |
|  | | | | | | | | | | | | | | | | | | | | | | | |
| **ID** | **Name** | **Z-Score*** | **MCC** | **DMNC** | | **MNC** | | **Degree** | | **EPC** | | **BottleNeck** | | **EcCentricity** | | **Closeness** | | **Radiality** | | **Betweenness** | | **Stress** | |
| KIF11 | Kinesin family member 11 | 4.80 | 1 | 116 | 13 | | 13 | | 36 | | 16 | | 1 | | 19 | | 25 | | 41 | | 33 | |  |
| CDK1 | Cyclin dependent kinase 1 | 5.86 | 1 | 312 | | 1 | | 1 | | 1 | | 2 | | 1 | | 1 | | 1 | | 5 | | 2 | |
| MCM5 | Minichromosome maintenance complex component 5 | 4.56 | 1 | 126 | | 20 | | 19 | | 33 | | 61 | | 1 | | 20 | | 21 | | 23 | | 22 | |
| BRCA1 | BRCA1 DNA repair associated | 6.26 | 1 | 330 | | 2 | | 2 | | 5 | | 3 | | 1 | | 3 | | 4 | | 6 | | 4 | |
| PCNA | Proliferating cell nuclear antigen | 7.94 | 1 | 228 | | 9 | | 8 | | 16 | | 61 | | 1 | | 7 | | 7 | | 21 | | 10 | |
| MCM4 | Minichromosome maintenance complex component 4 | 7.06 | 1 | 74 | | 21 | | 21 | | 24 | | 24 | | 1 | | 30 | | 40 | | 97 | | 68 | |
| RAD51 | RAD51 recombinase | 7.45 | 1 | 206 | | 12 | | 11 | | 12 | | 120 | | 1 | | 11 | | 9 | | 28 | | 15 | |
| FEN1 | Flap structure-specific endonuclease 1 | 3.00 | 1 | 128 | | 15 | | 15 | | 9 | | 84 | | 1 | | 23 | | 27 | | 87 | | 45 | |
| TPX2 | TPX2 microtubule nucleation factor | 4.71 | 1 | 59 | | 33 | | 34 | | 30 | | 61 | | 1 | | 39 | | 43 | | 139 | | 96 | |
| PCLAF | PCNA clamp associated factor | 4.99 | 1 | 45 | | 64 | | 63 | | 58 | | 42 | | 1 | | 52 | | 49 | | 89 | | 76 | |
| **Abbreviations:** Percolated Component (EPC), Maximum Neighborhood Component (MNC), Density of Maximum Neighborhood Component (DMNC), Maximal Clique Centrality (MCC).  *Expression level compared to healthy controls, following P<0.05 corrected by Benjamini-Hochberg False Discovery Rate. | | | | | | | | | | | | | | | | | | | | | | | |

| **Table S4.** Top 10 hub genes according to 11 topological algorithms ranked in the protein-protein interaction (PPI) network of cervical cancer (CC) differentially expressed genes. Numbers represent score. | | | | | | | | | | | | | |
| --- | --- | --- | --- | --- | --- | --- | --- | --- | --- | --- | --- | --- | --- |
|  | | | | | | | | | | | | | |
| **ID** | **Name** | **Z-Score*** | **MCC** | **DMNC** | **MNC** | **Degree** | **EPC** | **BottleNeck** | **EcCentricity** | **Closeness** | **Radiality** | **Betweenness** | **Stress** |
| AURKA | Aurora kinase A | 4.51 | 1 | 562 | 16 | 16 | 5 | 92 | 1 | 18 | 21 | 37 | 25 |
| PCNA | Proliferating cell nuclear antigen | 7.19 | 1 | 652 | 19 | 18 | 15 | 92 | 1 | 36 | 55 | 49 | 41 |
| CDK1 | Cyclin dependent kinase 1 | 8.80 | 1 | 965 | 3 | 3 | 1 | 4 | 1 | 4 | 6 | 10 | 6 |
| MCM4 | Minichromosome maintenance complex component 4 | 6.54 | 1 | 239 | 36 | 36 | 21 | 13 | 1 | 109 | 158 | 251 | 163 |
| BRCA1 | BRCA1 DNA repair associated | 9.51 | 1 | 985 | 6 | 6 | 12 | 29 | 1 | 11 | 11 | 14 | 12 |
| MCM5 | Minichromosome maintenance complex component 5 | 6.67 | 1 | 344 | 38 | 38 | 35 | 173 | 1 | 80 | 107 | 171 | 113 |
| CDC20 | Cell division cycle 20 | 7.86 | 1 | 512 | 9 | 9 | 6 | 473 | 1 | 25 | 36 | 73 | 42 |
| RAD51 | RAD51 recombinase | 5.54 | 1 | 516 | 23 | 24 | 16 | 473 | 1 | 28 | 31 | 76 | 43 |
| KIF23 | Kinesin family member 23 | 8.51 | 1 | 181 | 64 | 66 | 39 | 52 | 1 | 155 | 202 | 262 | 216 |
| KIF11 | Kinesin family member 11 | 8.83 | 1 | 358 | 29 | 29 | 14 | 473 | 1 | 70 | 112 | 83 | 75 |
| **Abbreviations:** Percolated Component (EPC), Maximum Neighborhood Component (MNC), Density of Maximum Neighborhood Component (DMNC), Maximal Clique Centrality (MCC).  *Expression level compared to healthy controls, following P<0.05 corrected by Benjamini-Hochberg False Discovery Rate. | | | | | | | | | | | | | |
