# Supplementary material for: PCNA in Cervical Intraepithelial Neoplasia and Cervical Cancer: An Interaction Network Analysis of Differentially Expressed Genes
Source: Front Oncol. 2021 Nov 26;11:779042. doi: 10.3389/fonc.2021.779042 (PMC8661029; doi:10.3389/fonc.2021.779042)

Supplementary Figures

Figure S1. (A) Protein-protein interaction network and **(B)** clustering modules of differentially expressed genes from cervical epithelial samples in patients with CIN. PPI network: green nodes, Cluster 1: yellow nodes, Cluster 2: blue nodes. CIN: Cervical intraepithelial neoplasia.

A

B

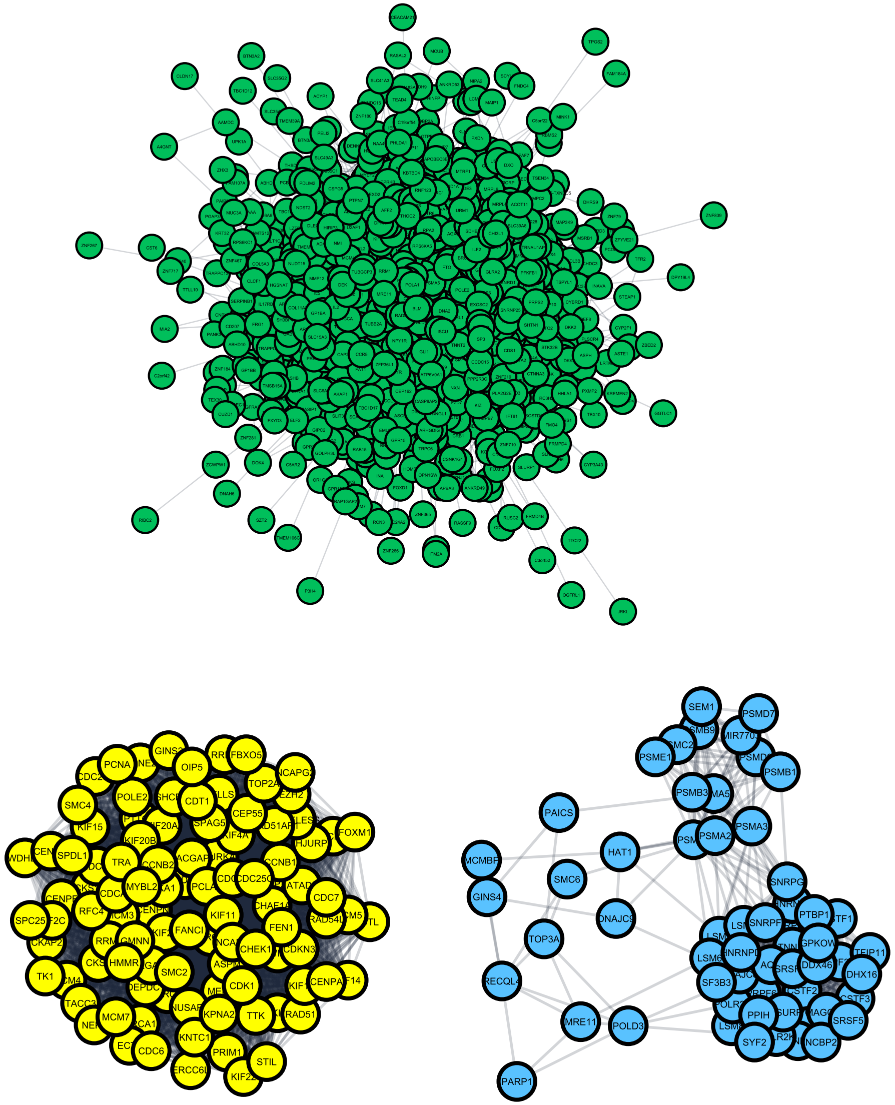

Figure S2. (A) Protein-protein interaction network and **(B)** clustering modules of differentially expressed genes from cervical epithelial samples in patients with CC. PPI network: red nodes, Cluster 1: yellow nodes, Cluster 2: blue nodes, Cluster 3: purple nodes. CC: Cervical cancer.

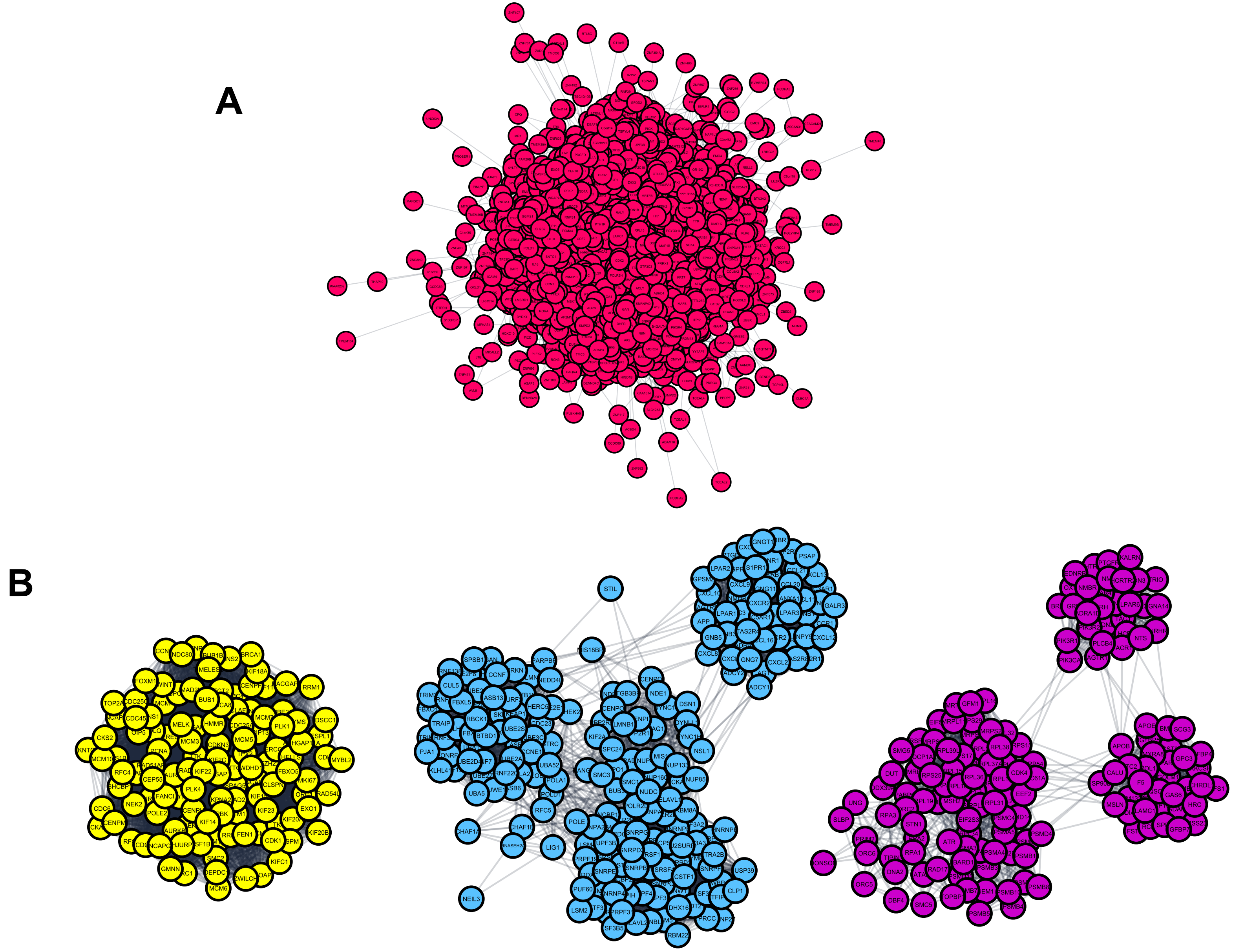

Figure S3. Overall expression (transcripts per million) of PCNA based on the TCGA, in pan-cancer patients. Significance between tumour state (red) and healthy controls (blue) was determined using a P<0.05 and |log2 fold change|>2. PCNA: Proliferating cell nuclear antigen. ACC: Adrenocortical carcinoma; BLCA: Bladder Urothelial Carcinoma; BRCA: Breast invasive carcinoma; CESC: Cervical squamous cell carcinoma and endocervical adenocarcinoma; CHOL: Cholangio carcinoma; COAD: Colon adenocarcinoma; ESCA: Esophageal carcinoma; HNSC: Head and Neck squamous cell carcinoma; KICH: Kidney Chromophobe; KIRC: Kidney renal clear cell carcinoma; KIRP: Kidney renal papillary cell carcinoma; LIHC: Liver hepatocellular carcinoma; LUAD: Lung adenocarcinoma; LUSC: Lung squamous cell carcinoma; PAAD: Pancreatic adenocarcinoma; PCPG: Pheochromocytoma and Paraganglioma; PRAD: Prostate adenocarcinoma; READ: Rectum adenocarcinoma; SARC: Sarcoma; SKCM: Skin Cutaneous Melanoma; STAD: Stomach adenocarcinoma; TCGA: The Cancer Genome Atlas; THCA: Thyroid carcinoma; THYM: Thymoma; UCEC: Uterine Corpus Endometrial Carcinoma.

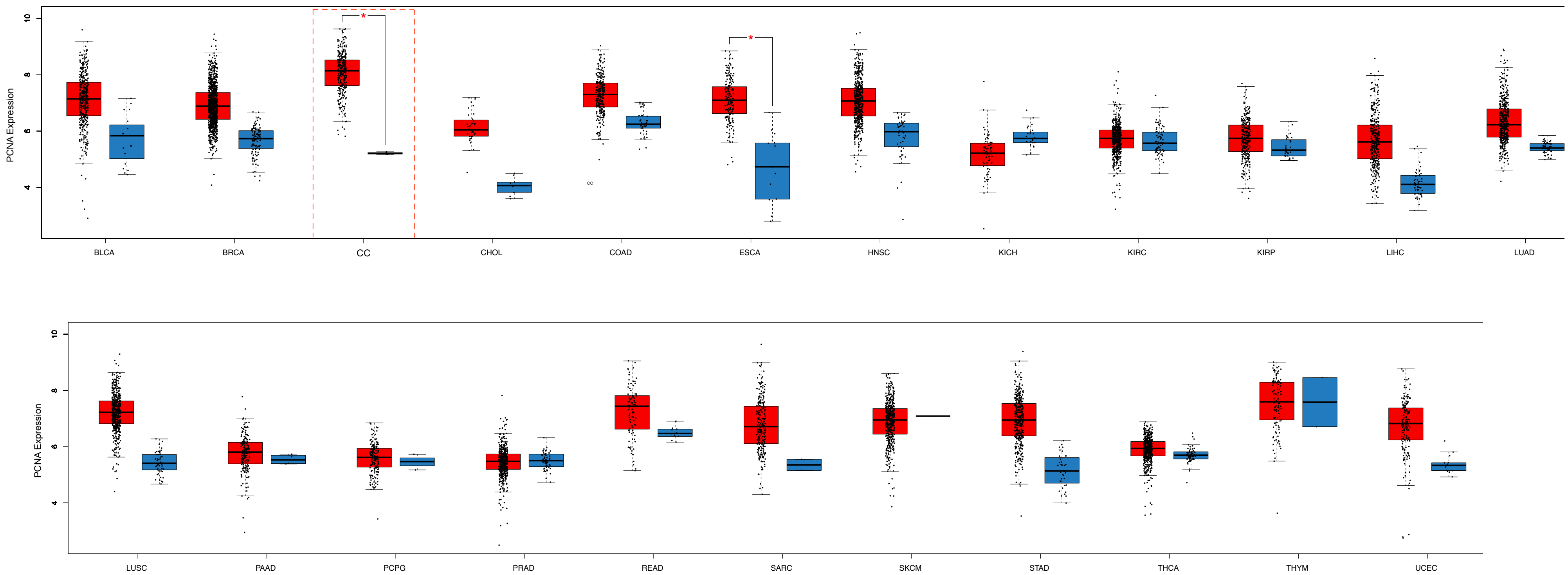

Supplement: Supplementary file 2 [file DataSheet_2.pdf]
